# Nitrate fertilisation does not enhance CO_2_ responses in two tropical seagrass species

**DOI:** 10.1038/srep23093

**Published:** 2016-03-15

**Authors:** Y. X. Ow, N. Vogel, C. J. Collier, J. A. M. Holtum, F. Flores, S. Uthicke

**Affiliations:** 1 College of Marine and Environmental Science, James Cook University, Townsville Queensland 4811, Australia; 2 Australian Institute of Marine Science, Townville MC Queensland 4810, Australia; 3 Centre for Tropical Water & Aquatic Ecosystem Research (TropWATER), James Cook University, Cairns, Queensland 4870, Australia; 4 Experimental Marine Ecology Laboratory, Department of Biological Sciences, National University of Singapore, 14 Science Drive 4, Blk S3, #02-05, 117543, Singapore

## Abstract

Seagrasses are often considered “winners” of ocean acidification (OA); however, seagrass productivity responses to OA could be limited by nitrogen availability, since nitrogen-derived metabolites are required for carbon assimilation. We tested nitrogen uptake and assimilation, photosynthesis, growth, and carbon allocation responses of the tropical seagrasses *Halodule uninervis* and *Thalassia hemprichii* to OA scenarios (428, 734 and 1213 μatm *p*CO_2_) under two nutrients levels (0.3 and 1.9 μM NO_3_^−^). Net primary production (measured as oxygen production) and growth in *H. uninervis* increased with *p*CO_2_ enrichment, but were not affected by nitrate enrichment. However, nitrate enrichment reduced whole plant respiration in *H. uninervis*. Net primary production and growth did not show significant changes with *p*CO_2_ or nitrate by the end of the experiment (24 d) in *T. hemprichii*. However, nitrate incorporation in *T. hemprichii* was higher with nitrate enrichment. There was no evidence that nitrogen demand increased with *p*CO_2_ enrichment in either species. Contrary to our initial hypothesis, nutrient increases to levels approximating present day flood plumes only had small effects on metabolism. This study highlights that the paradigm of increased productivity of seagrasses under ocean acidification may not be valid for all species under all environmental conditions.

Ocean acidification (OA) increases seawater carbon dioxide (CO_2_) concentration and alters the relative proportion of dissolved inorganic carbon (DIC) species in seawater^1^. Seawater concentrations of CO_2_ and bicarbonate were projected to rise by 250% and 24%, respectively, up from current levels of 8 and 1650 μmol kg^−1^ seawater by the end of the century^2^. Seagrass productivity, thought to be limited by current seawater DIC composition, could benefit from the increased availability of carbon^3^. Studies have shown that photosynthetic rates of most seagrasses were enhanced by elevated partial pressure of CO_2_ (*p*CO_2_)^4^,^5^, which is the preferred DIC species^3^,^6^. Carbon fixed in the leaves through photosynthesis has a number of sinks and therefore, under increased *p*CO_2_, growth, respiration, storage, biomass and reproductive output may be increased^4^,^5^,^7^,^8^,^9^.

The paradigm that OA benefits seagrass meadow productivity assumes that other environmental parameters, such as nutrient levels are not co-limiting productivity^5^,^6^. In terrestrial plants, nutrient availability can affect responses to elevated CO_2_; they initially respond by increasing productivity and growth but photosynthesis and growth are subsequently downregulated as nitrogen becomes limited^10^. Coastal seagrass systems can be subjected to fluctuations in water column nutrient levels^11^. While strong and sustained nutrient enrichment can stimulate the growth of competing macroalgae and epiphytes and in turn inhibit seagrass growth^12^, moderate increases in nutrients can promote seagrass growth, which demonstrates nutrient limitation^13^,^14^.

Responses to elevated *p*CO_2_ are affected by nutrient availability because carbon and nitrogen metabolism are strongly coupled^15^. Nitrate and nitrogen metabolites regulate processes such as photosynthesis^16^, organic acid synthesis and starch accumulation^17^; leading some authors to speculate that moderate increases in dissolved inorganic nitrogen (DIN) may augment CO_2_ responses in tropical seagrasses^9^,^18^. In marine macroalgae, productivity responses to CO_2_ enrichment were enhanced under increased nutrient availability when compared to non-enriched nutrient conditions^19^,^20^,^21^.

Energy stored in carbon metabolites plays a role in regulating the uptake and incorporation of nitrogen^22^. Therefore, the demand for nitrogen could be affected by the rate of carbon assimilation, and also by CO_2_ enrichment^10^. With increased nutrient availability, marine macroalgae in enriched *p*CO_2_ conditions increased photosynthetic efficiency^19^,^21^, growth^19^ and nitrogen uptake and assimilation^20^, observations consistent with an increase in the demand for nitrogen driven by enhanced productivity.

Nitrogen incorporation involves both the uptake and assimilation of nitrogen species^22^. Both uptake and assimilation are inducible processes that may reflect instantaneous nitrogen demand in the plant^22^. For seagrasses, inorganic nitrate and ammonium are considered the most significant sources of nitrogen, supplying over 90% of externally acquired nitrogen^22^,^23^. Sediment pore-water can potentially supply the majority of nitrogen for seagrass as the sediment contains higher concentrations of nitrogen than the water column does, but seagrasses will rapidly absorb DIN from the water column^24^. Furthermore, the uptake affinity (K_m_) of leaves is greater than that of rhizomes, meaning that a small increase in supply to the water column will trigger rapid uptake^24^. Nitrogen assimilation involves the enzymatic conversion of nitrate to nitrite by nitrate reductase (NR), and ammonium to glutamine through the glutamine synthetase (GS)/glutamate synthase pathway^22^. The activities of NR and GS, key in amino acids synthesis^25^, occur primarily in leaves and to a much smaller degree, in the rhizomes and roots^26^. Therefore, increasing external inorganic nitrogen may promote nitrogen uptake and assimilation in seagrasses^25^,^26^.

The internal partitioning of fixed carbon to sink tissues and processes is affected by nitrogen availability^9^,^18^ and other environmental cues^27^,^28^. The flux of fixed carbon in each tissue organ is controlled by key enzymes. For example, sucrose-phosphate synthase (SPS) in mature photosynthetic leaves primes the conversion of carbon into sucrose, which is subsequently transported to sinks^15^. The import of sucrose into sinks is controlled by sucrose synthase (SS). Under CO_2_ enrichment, reduced nitrogen availability could direct more carbon into below-ground biomass for storage, reducing nutrient imbalances in the leaves^29^.

The effects of nutrient enrichment on response to increasing *p*CO_2_ are likely to be greatest in regions where DIN is relatively low. We hypothesized that 1) *p*CO_2_ and nitrate enrichment can have additive effects on seagrass productivity and biomass and 2) *p*CO_2_ enrichment drives nitrogen demand. To test this, we increased DIN and *p*CO_2_ levels in seawater, to simulate DIN levels in flood plumes (average 2.20 μM across the Great Barrier Reef) and predicted end-of-century levels under RCP 2.6 and RCP 8.5 CO_2_ emission scenarios^30^. To allow wider inference we examined common species with different growth and storage strategies, the fast-growing species *Halodule uninervis* and the slow-growing species *Thalassia hemprichii*^31^. Both species contribute to the productivity and resilience of tropical seagrass meadows over different successional stages. Assessment of growth and productivity permitted us to test the first hypothesis, and measurement of nitrogen incorporation processes (uptake and assimilation) enabled testing of the second hypothesis.

## Results

### Experimental parameters

Water temperature (daily range 27.8–29.8 °C) and salinity (34.6–34.9) were similar between experimental tanks and throughout the experiment (Table 1). Carbonate system parameters of the enriched *p*CO_2_ treatments remained well within the target range of 428, 734 and 1213 μatm for the three treatments (Table 1). Average ammonium (0.59 μM; S.D. = 0.28 μM) and phosphate (0.05 μM; S.D. = 0.02 μM) concentrations were similar between treatments. Nitrate concentration was 0.29 ± 0.18 μM (S.D.) and 1.91 ± 0.33 μM (S.D.) in ambient and nutrient enriched treatments respectively.

### Productivity and growth

**Figure 1:**
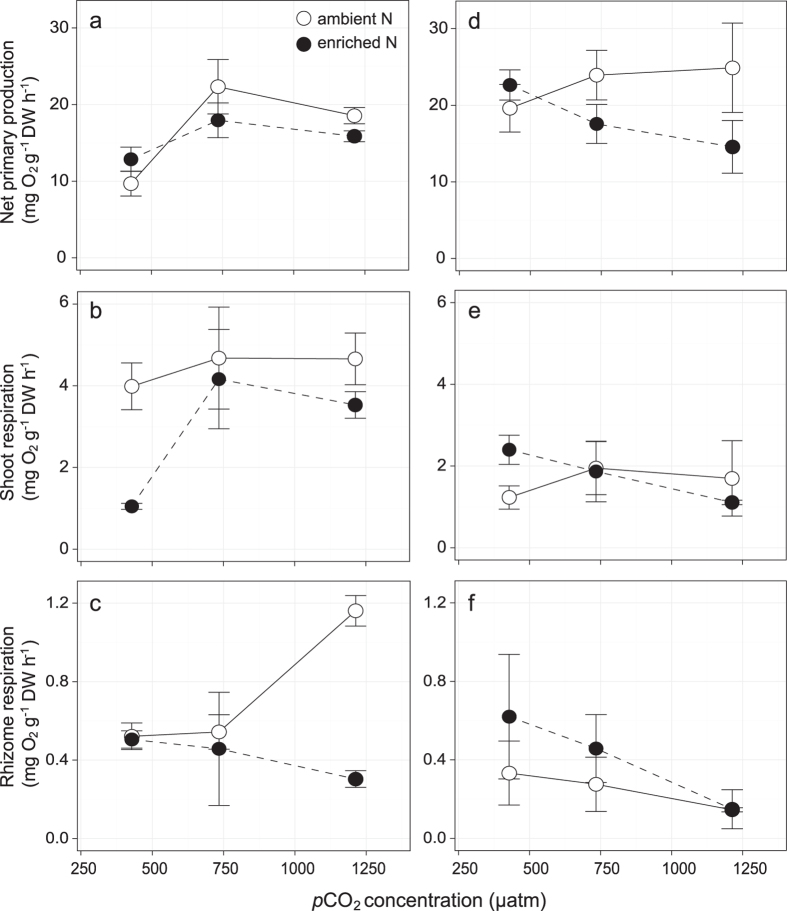
Net primary production and respiratory responses of (a–c) *H. uninervis* and (d–f) *T. hemprichii* measured after 22 days exposure to treatment. Values are average ± S.E. N = 3.

In *H. uninervis*, net primary production increased with *p*CO_2_ levels (LME: *P* = 0.049) (Fig. 1; Table 2). The linear model predicted an increase of 1.071 mg O_2 _g^−1^ DW h^−1^ in net primary production for every 100 μatm rise in *p*CO_2_. There was no effect of nitrate enrichment on primary production (Table 2). Leaf respiration was not affected by *p*CO_2_ levels, but decreased by 34% with nitrate enrichment (LME: *P* = 0.025) (Fig. 1; Table 2). Rhizome respiration responses to *p*CO_2_ depended on nitrate enrichment (LME *p*CO_2_ × nitrate interaction: *P* = 0.009) (Fig. 1; Table 2). Under ambient DIN conditions, rhizome respiration increased with *p*CO_2_; under enriched DIN, rhizome respiration decreased with *p*CO_2_ (Fig. 1).

**Figure 2:**
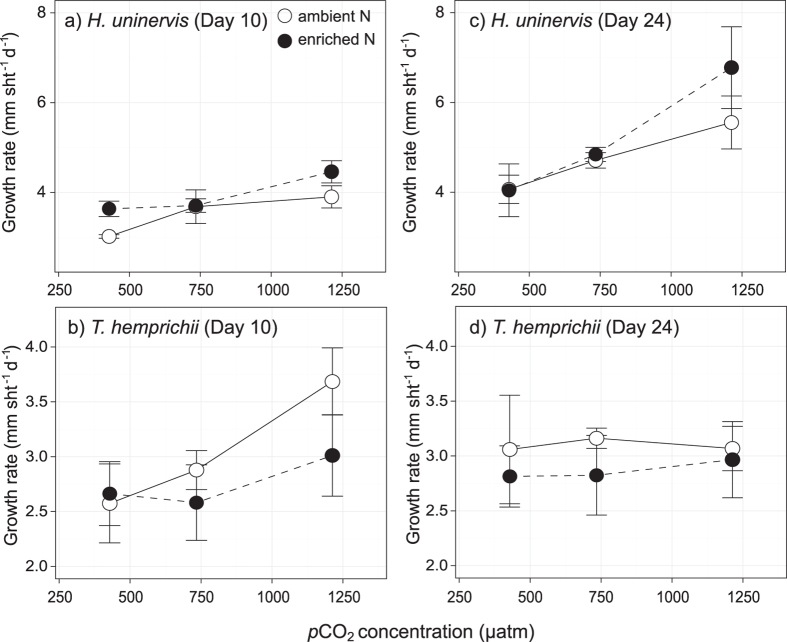
Growth rates of (a,c) *H. uninervis* and (b,d) *T. hemprichii* after 10 and 24 days exposure to treatments. Values are average ± S.E. N = 3.

Growth rates of *H. uninervis* shoots increased with *p*CO_2_ enrichment after 10 days (LME: *P* = 0.006) (Fig. 2; Table 2). At day 10, growth rates increased from 3.3 mm shoot^−1^ day^−1^ in control *p*CO_2_ aquaria (428 μatm) to 4.2 mm shoot^−1^ day^−1^ in high *p*CO_2_ aquaria (1213 μatm). The enhancement of growth rates with *p*CO_2_ was sustained after 24 days (LME: *P* = 0.001) as growth rates in control *p*CO_2_ aquaria were 4.1 mm shoot^−1^ day^−1^, while those in high *p*CO_2_ aquaria were elevated by 52% (6.2 mm shoot^−1^ day^−1^). There was no significant effect of nitrate enrichment on growth (Fig. 2; Table 2). Shoot growth of *H. uninervis* in the source meadow at day 13–17 of the experiment was in a similar range (7.0 mm shoot^−1^ day^−1^; S.E. = 1.24 mm shoot^−1^ day^−1^).

Net primary production in *T. hemprichii* did not increase with *p*CO_2_ or nitrate enrichment (Fig. 1; Table 2). In addition, no significant changes in leaf and rhizome respiration with *p*CO_2_ and nitrate enrichment were detected.

In *T. hemprichii*, at day 10, leaf growth rates responded to *p*CO_2_ enrichment and no effect of nitrate enrichment was detected (LME: *p*CO_2_ - *P* = 0.024; nitrate - *P* = 0.252) (Fig. 2; Table 2). Growth rates increased by 28% with *p*CO_2_ enrichment. By day 24, no change in growth rate to *p*CO_2_ or nitrate was detected (Fig. 2; Table 2). Overall, growth of *T. hemprichii* in the experimental aquaria (global average = 2.98 mm shoot^−1^ day^−1^; S.E. = 0.12 mm shoot^−1^ day^−1^) was lower than that measured in the source meadow (5.95 mm shoot^−1^ day^−1^; S.E. = 0.57 mm shoot^−1^ day^−1^).

### Carbohydrates translocation and storage

For both *H. uninervis* and *T. hemprichii*, *p*CO_2_ manipulation did not affect sucrose-phosphate synthase (SPS) and sucrose synthase (SS) activity indicative of carbohydrate translocation (Table 2). Nutrient enrichment reduced SPS activity in *H. uninervis* leaves (LME: *P* = 0.040) (Table 2), but overall the effects were of limited consequence for our hypotheses (see Supplementary Fig. 1). Non-structural carbohydrates in *H. uninervis* and *T. hemprichii* rhizomes showed no change to *p*CO_2_ and nitrate enrichment (Table 2).

### Nitrogen uptake and assimilation

**Figure 3:**
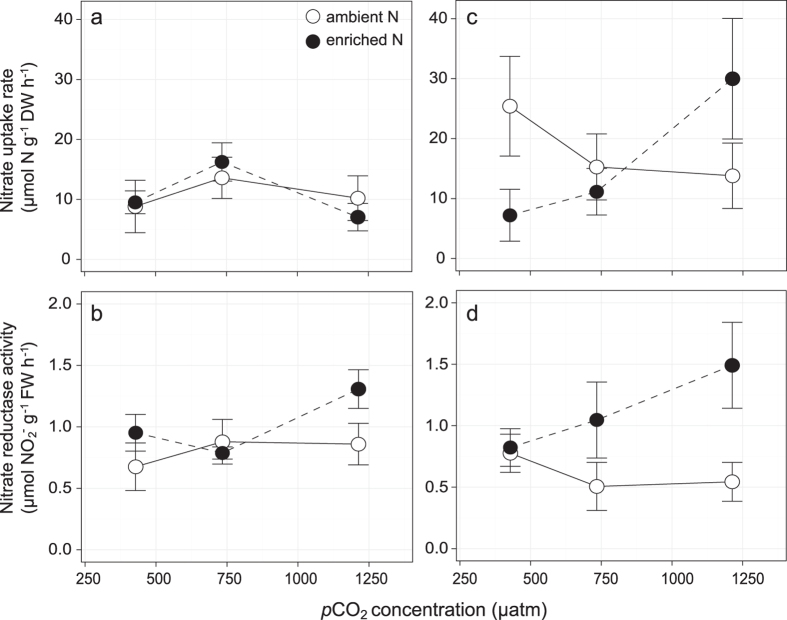
Nitrate incorporation (uptake and assimilation) in leaves of (a,b) *H. uninervis* and (c,d) *T. hemprichii* across a range of *p*CO_2_ concentrations. Values are average ± S.E. N = 3.

In *H. uninervis*, leaf uptake of nitrate, determined by ^15^N incorporation, did not vary with *p*CO_2_ or nitrate enrichment in *H. uninervis* (10.91 μmol N g^−1^ DW h^−1^; S.E. = 1.35 μmol N g^−1^ DW h^−1^) (Fig. 3; Table 3). No significant changes in nitrogen assimilation (enzymatic activity) in *H. uninervis* with *p*CO_2_ and nitrate enrichment were detected (Table 3). Furthermore, there were no changes in leaf tissue nutrient content (means ± S.E.: C–41 ± 0.2%; N–2.5 ± 0.03%; C:N–16.3 ± 0.2) (Table 2).

In *T. hemprichii*, nitrate uptake was increased with *p*CO_2_, but only in the nitrate enriched treatment (LME *p*CO_2_ × nitrate interaction: *P* = 0.017) (Fig. 3; Table 3). Nitrate uptake rates increased by 117% at the highest *p*CO_2_ relative to ambient levels. In *T. hemprichii* leaves, NR activity was higher with nitrate enrichment (linear model *P* = 0.019) but was not affected by *p*CO_2_ levels (Table 3). NR activity in *T. hemprichii* leaves in ambient seawater (0.61 μmol NO_2 _g^−1^ FW h^−1^; S.E. = 0.14 μmol NO_2 _g^−1^ FW h^−1^) was ~50% that in enriched nitrate conditions (1.12 μmol NO_2 _g^−1^ FW h^−1^; S.E. = 0.25 μmol NO_2 _g^−1^ FW h^−1^). GS activity in *T. hemprichii* leaves did not change significantly with *p*CO_2_ or nitrate (69.50 μmol g^−1^ FW h^−1^; S.E. = 5.06 μmol g^−1^ FW h^−1^) (Table 3). There were no significant changes in leaf carbon content (39 ± 0.3%), but there were marginal increase in leaf nitrogen (LME: *P* = 0.056) and significant reduction in C:N ratio (LME: *P* = 0.045) with nitrate enrichment in *T. hemprichii* (Table 3). Under ambient nitrate levels, nitrogen content and C:N were 2.7 ± 0.06% and 14.7 ± 0.27 respectively; with nitrate enrichment, nitrogen content was 2.8 ± 0.08% and C:N was 14.0 ± 0.41 (means ± S.E.).

## Discussion

This study aimed to test whether seagrass productivity is affected by *p*CO_2_ and nitrate (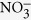) enrichment, and whether *p*CO_2_ drives the demand for nitrogen in seagrasses. In *H. uninervis*, net primary production (NPP) and growth rates increased with higher *p*CO_2_ but were not affected by nitrate enrichment. However, in *T. hemprichii*, NPP and growth were not affected by either *p*CO_2_ or nitrate enrichment. In *H. uninervis*, *p*CO_2_ enrichment did not increase nitrate uptake or assimilation while nitrate uptake was higher in CO_2_-enriched (simulating end of century RCP 8.5 emission scenario)^30^ *T. hemprichii*. In addition, nitrate enrichment (1.9 μM compared to 0.3 μM in ambient) raised leaf nitrate reductase (NR) activity in *T. hemprichii*. Therefore, productivity responses to *p*CO_2_ and nitrate enrichment varied between species with different growth strategies.

*H. uninervis* and *T. hemprichii* differed in productivity responses to *p*CO_2_ enrichment after 24 days exposure. In *H. uninervis*, NPP increased by 1.1 units for every 100 μatm rise in *p*CO_2_, an increase slightly higher than the 0.9 units measured in the same species by Ow *et al.*^4^. Other fast-growing seagrass species that have increased photosynthetic rates with *p*CO_2_ enrichment include *Z. marina* (250% increase at pH 6.2, relative to 338 μatm *p*CO_2_)^5^ and *Z. noltii* (34% increase at pH 7.9, relative to 360 μatm *p*CO_2_)^32^. Leaf growth rates in *H. uninervis* were also enhanced in *p*CO_2_ enriched treatments, with the highest leaf growth rates [6.2 ± 0.40 (S.E.) mm shoot^−1^ day^−1^] being slightly lower than that measured in the field [7.0 ± 1.24 (S.E.) mm shoot^−1^ day^−1^]. Aquaria experiments may impose potential artefacts on leaf growth due to transplantation stress, which were minimised by allowing for acclimation prior to experiments. However, as described below, light levels within experimental tanks, which were lower than that of nearby shallow reef systems, most likely explained the lower growth rates in aquaria.

In *T. hemprichii*, *p*CO_2_ enrichment had no effect on NPP and growth rates after three weeks, in contrast to previous work on this species^4^,^7^. Jiang *et al.*^7^ studied *T. hemprichii* from a nutrient-enriched meadow (0.8–4.6 μM NO_3_^−^ + NO_2_^−^)^33^ and exposed to much higher CO_2_ concentrations (25–1005 μM) compared to the present study (19–31 μM). *T. hemprichii* grown under high nitrogen might have utilised its pre-existing nutrients store^22^ to supplement a rapid growth increase during strong CO_2_ enrichment^7^. In the present study, *T. hemprichii* productivity did not appear to be nitrogen-limited (discussed below), indicating that light levels in experimental tanks, or phosphate availability in carbonate sediments^34^ could have limited its growth response. Interestingly, leaf growth of *T. hemprichii* showed a transient rise with *p*CO_2_ at day 10, but subsequently stabilised. This growth response to initial (short-term) *p*CO_2_ exposure has been reported for *T. hemprichii* after 14 days of exposure^4^. However, NPP measured at the end of the experiment (22 days) suggest a downregulation in response to *p*CO_2_ over time.

Nitrate addition did not increase NPP in *H. uninervis*. This was despite respiration rates of the rhizome-root complex in enriched *p*CO_2_ being lowered with nitrate enrichment. Given the relatively large proportion of below-ground biomass for this species^35^, a reduction in rhizome-root respiration could be substantial for improving carbon use^36^. In the present study, lower sucrose phosphate synthase (SPS) activity^15^,^27^ in *H. uninervis* exposed to nitrate enrichment suggested a decline in the export of fixed carbon from leaves, potentially due to reduced metabolic demand in the rhizome-root biomass^8^. Further quantification of nitrate uptake rates and of the activities of the key enzymes in the nitrogen assimilation pathway, nitrate reductase and glutamine synthetase^22^,^25^, revealed no effect of nitrate enrichment on nitrogen incorporation in *H. uninervis*.

Productivity in *T. hemprichii* did not increase with nitrate enrichment, even though nitrate enrichment increased nitrate uptake at high *p*CO_2_ and assimilation in the leaves of *T. hemprichii*. Increased nitrate uptake and assimilation under water-column nitrate enrichment could be advantageous for seagrasses acclimatised to growing in a low-nitrogen environment^15^. This allows the plant to sequester and store nitrogen rapidly when it becomes available. Higher nitrogen content and a lowered C:N ratio were observed in nitrate-enriched *T. hemprichii* leaves. Therefore, nitrate enrichment appeared to have a greater influence on nitrogen incorporation in *T. hemprichii* than *H. uninervis*.

Overall there was no evidence in the present study that nitrate enrichment enhanced productivity responses to *p*CO_2_ for either species. This was surprising as nitrogen had been suggested^32^ and shown to limit the productivity of marine macrophytes to *p*CO_2_ enrichment^21^ in subtidal rocky habitats. The experiment duration might not have been long enough for *p*CO_2_ enrichment to induce a significant change in nitrogen demand (24 days vs 5 months^32^), which may still be covered by pre-existing nitrogen-resources. Previous work reported increases in leaf tissue carbon-to-nitrogen (C:N) ratios in CO_2_ enriched seagrasses^7^,^37^, which suggested nitrogen limitation in these plants. However, C:N ratios in both *H. uninervis* and *T. hemprichii* here revealed no evidence that *p*CO_2_ enrichment led to the seagrasses requiring more nitrogen. In the Great Barrier Reef (GBR) region, seagrass growth was limited by nitrogen at some sites^13^,^38^. In the present study, leaf nitrogen content and C:N ratios of *H. uninervis* (N = 2.53%; C:N = 16.3) and *T. hemprichii* (N = 2.75%; C:N = 14.4) were similar to previous values measured in GBR seagrasses^39^. These were well above the values assumed to indicate nitrogen limitation (N = 1.8%; C:N = 20)^39^,^40^ and suggest that the two species were not nitrogen limited. DIN levels in sediment pore-water and that adsorbed to sediments were not quantified here, but typical concentrations can be 200 times higher than in the water column^24^. Thus sediment pore-water may have supplied sufficient DIN to maintain productivity rates measured here. Another possible explanation for apparent nutrient sufficiency (C:N < 20)^41^ is that light levels during the experiment, averaging 9 mol m^−2 ^d^−1^, were low compared to longer-term monitoring from shallow seagrass meadows in far north Queensland which typically reach 15–20 mol m^−2 ^d^−1^. Furthermore light levels dropped in the region of the study site (Cape York) in early 2014^39^. Lowered levels of natural light, relative to the typical levels available^39^, may also explain the limited productivity responses to *p*CO_2_.

Carbon dioxide enrichment did not drive nitrogen demand in *H. uninervis* and *T. hemprichii*. In other marine macrophytes, CO_2_ enrichment was shown to increase nitrate reductase activity^32^,^42^. Here, increased CO_2_ availability did not affect nitrate uptake and assimilation (measured as nitrate reductase and glutamine synthetase activity) in *H. uninervis*, whereas the effect was dependent on nitrate enrichment in *T. hemprichii*. This is interesting as water column DIN concentrations at northern mid-shelf GBR (e.g. Lizard Island) are typically lower than that at inshore reefs^43^, where the majority of seagrass grows^44^. Perhaps experiments on longer time-scales are needed to evaluate the effects of nitrogen availability on productivity, as seagrasses possess mechanisms to improve nitrogen-use efficiency, likely through recycling or re-allocation of nitrogen within the plant^24^. At natural CO_2_ seeps with elevated *p*CO_2_, no difference in tissue nutrients were found between seagrasses growing around, and away from the CO_2_ seeps, suggesting CO_2_-induced nitrogen limitation was not present^45^. Continual flux in nutrients in coastal habitats, supplemented by nitrogen fixation in the sediments^46^, may enable seagrasses to be more productive without facing nitrogen limitation with future OA.

In conclusion, the tropical seagrasses, *H. uninervis* and *T. hemprichii,* did not appear to be strongly nitrogen limited despite being collected from a mid-shelf reef where ambient water column nitrogen concentrations were low (0.13 μmol DIN). Consequently, nitrate fertilization of the water column did have some effect on nitrate uptake rates, but did not enhance seagrass productivity or leaf growth rates. Furthermore, in contrast to our initial hypothesis, responses to *p*CO_2_ enrichment, simulating future ocean acidification scenarios, were also unaffected by nitrate fertilisation. To better reconcile the effects of nutrient enrichment on seagrass CO_2_ responses with previous studies, there is the need to account for differences in background light, nutrient levels and durations between experiments. This helps to circumvent the current experimental limitations in expanding our findings to a wider environment. Ocean acidification can also promote the growth of epiphytic filamentous algae, outweighing the influence of nutrient addition on seagrass epiphytes^47^. Nutrient enrichment could encourage a shift in the dominance of submerged vegetation, from seagrasses to fast-growing macroalgae and phytoplankton, such as that observed in habitats exposed to eutrophication^48^. Hence, while seagrass meadows may potentially flourish in a future where the oceans are enriched in CO_2_, ecological effects of ocean acidification and nutrient fertilisation, such as competition from macroalgae and epiphytes, may outweigh gains to seagrass productivity.

## Methods

### Plant collection and experimental setup

The experiment was carried out at Lizard Island, GBR, Australia, in March 2014. *Halodule uninervis* was collected from an intertidal meadow and *Thalassia hemprichii* from the subtidal zone (2–3 m depth) of One Tree Coconut beach (14° 41.370’S, 145° 27.392’E) following protocols described in Ow *et al.*^4^. Seagrasses were potted up within 48 h of collection in the same sediments from their source meadows (*H. uninervis* in 20:80 carbonate sand:site mud mixture, *T. hemprichii* in carbonate sand). Potted seagrasses were stored in outdoor flow-through aquaria (50 L) for three to six days prior to the initiation of the experiment. Experimental treatments consisted of three *p*CO_2_ levels (ambient ~428 μatm, moderate ~734 μatm and high ~1213 μatm *p*CO_2_) and two nitrate treatments (ambient ~0.3 μM and enriched ~1.9 μM) crossed in a fully factorial design. Each treatment comprised of three replicate 25 L aquaria leading to a total of eighteen aquaria, supplied with seawater at 24 L h^−1^ directly from the adjacent lagoon. Two sub-replicate pots of each species were placed in each aquarium. The aquaria were situated outdoors under a solid translucent roof, which attenuated 50% of down-welling light. 2π light loggers (Odyssey, New Zealand) were randomly allocated to aquaria to record photosynthetically active radiation (PAR). Over the course of the experiment, the net daily PAR in aquaria ranged from 1.2–5.2 mol m^−2 ^d^−1^, averaging 3.8 mol m^−2 ^d^−1^. Mid-day maximum PAR averaged to 480 μmol m^−2 ^s^−1^. Treatments were randomised between the aquaria to eliminate any potential environmental effects within the set-up area. The experiment ran for 24 days before it had to be terminated due to an approaching cyclone.

*p*CO_2_ concentrations were manipulated by injecting different amounts of CO_2_ gas into sump tanks. pH levels in the sump tanks were monitored with six potentiometric sensors (±0.01 pH unit) calibrated on the NIST (National Institute of Standards and Technology) scale as a proxy to control for CO_2_ input. The sensors provide feedback to a control system that regulates pH levels via CO_2_ gas injection (AquaMedic, Germany)^4^. We recognise that over natural seagrass meadows, seawater pH fluctuates and does not have a set point. However, such fluctuations are hard to emulate while controlling for *p*CO_2_ concentrations with our current set-up. Hence, *p*CO_2_ concentrations were controlled using fixed pH levels instead. Seawater *p*CO_2_ concentrations in mid-shelf reefs, such as Lizard Island in the GBR averaged about 380 μatm (1 S.D. = 15 μatm)^49^ during the dry season from 2011–2013. During the wet season, when the present experiment was conducted, *p*CO_2_ concentrations tend to be higher (460 μatm; 1 S.D. = 33 μatm) than during the dry season^49^.

Across the Great Barrier Reef (GBR), DIN (nitrate, ammonium and nitrite) levels in the water column over seagrass meadows are relatively low, averaging 0.13 μM^11^. However, terrestrial run-off into coastal areas can deliver DIN loads that are an order of magnitude or more higher (1.54 to 7.02 μM, or 2.20 μM averaged across the GBR)^50^. Nitrate enrichment was achieved by dripping sodium nitrate solution (Sigma-Aldrich, Australia) into individual aquaria. Peristaltic pumps (Cole Palmer, USA) delivered 2 mM of NaNO_3_ solution into the individual aquaria at a rate of 0.5 ml min^−1^. Small aquaria pumps (Hailea, China) in each aquarium provided mixing.

### Seawater chemistry

pH_total_ in treatment tanks were monitored by spectrometric determination of m-cresol absorbance^51^, and additionally checked against TRIS seawater standard (A. G. Dickson, Scripps Institute of Oceanography, Batch 106). Weekly water samples were analysed for total alkalinity (A_T_) by gran titration with 0.5 M HCl on a Metrohm 855 titrosampler (Metrohm, Switzerland), and for total dissolved inorganic carbon (DIC) by acid titration on a VINDTA 3C. Carbonate system parameters were calculated using measured values of A_T_, DIC, temperature and salinity on CO2calc software^52^. Duplicate water samples for dissolved inorganic nutrient analysis were filtered through 0.45 μm cellulose acetate filters and stored at −20 °C before determination of seawater ammonium, nitrate, and phosphate concentrations according to standard procedures outlined in Ryle *et al.*^53^. Temperature in the treatment tanks was logged by HOBO tidbit loggers (Onset, USA) every 5 min. Salinity readings were taken from an IMOS weather buoy (Integrated Marine Observing System; www.aims.gov.au) situated in the lagoon.

### Productivity

After 22 days, photosynthetic and respiration rates were measured using the second youngest leaf of a shoot from each sub-replicate pot using optical oxygen sensors (“optodes”, PreSens, Germany) and a fiber-optic oxygen meter (PreSens Oxy 4, Germany). Respiration rates of below-ground rhizome with associated roots (~2.5 cm) from each pot were quantified similarly. Measurements were conducted in 70 mL chambers at constant 28 °C water temperature following procedures described in Ow *et al.*^4^. Respiration of the leaves and below-ground rhizome-roots were measured separately over a 20-min period in the dark while photosynthetic rates were measured on the same leaf at 400 μmol m^−2 ^s^−1^ PAR over 30 min. Plant material was dried (60 °C for 48 h) and weighed after incubation. Photosynthetic and respiration rates were normalised to the dry weight of the leaf and rhizome. Optodes were calibrated according to protocol described in Collier *et al.*^54^.

Growth rates were measured according to the method described in Short and Duarte^55^. At day 0 and day 14 of the experiment, all shoots were marked at the top of the bundle sheath with a needle. Length of new tissue growth was measured with vernier callipers regularly throughout the experiment, totalled and normalised to the number of shoots and days since marking. Growth rates of plants, from three separate plots in each source meadow, were also obtained using the same method from day 13 to day 17.

### Nitrogen uptake

Leaf nitrate uptake rates were estimated at the end of the experiment. Seagrass shoots were incubated in seawater enriched with ^15^N labelled potassium nitrate (atom% = 98; Novachem, Australia), and the final ^15^N in the leaf tissue was used to calculate the uptake of ^15^NO_3_^−^. Incubations were carried out on individual shoots in their pots, in their respective treatment tanks, via a method similar to that described in Prado *et al.*^56^. Individual shoots were enclosed within a plastic bag (~250 mL volume) fitted with a filter cassette and a plug that could be sealed. No leakage was detected when tested using a food dye. Potassium nitrate solution was injected into the chambers to achieve around 20% ^15^NO_3_^−^ enrichment of the initial ambient DIN concentration^57^. The shoots were incubated for one hour at ambient mid-day temperature (28 °C) and light (450 μmol m^−2 ^s^−1^). After one hour, the shoots were excised from the rhizomes and rinsed with deionized water to remove excess adherent label. Non-incubated leaf samples were collected from each tank to provide background leaf ^15^N levels for each species. Leaf material was processed and measured for total nitrogen content and atom% ^15^N according to method described in Takahashi *et al.*^45^. Uptake rates (μmol N g^−1^ dry weight h^−1^) of ^15^NO_3_^−^ were calculated following equations outlined in Nayar *et al.*^23^. The atom% ^15^N of ^15^N enriched seawater was calculated based on the amount of atom% 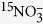 added and background DIN concentrations (assumed to reflect ^15^N concentration of atmospheric N ~ 0.37 atom% ^15^N).

### Nitrogen assimilation and carbon translocation

Plant material used for measuring nitrogen assimilation and carbon translocation (i.e. enzyme analyses), except for nitrate reductase (NR), were collected at the end of the experiment and stored in liquid nitrogen until analysis.

NR activity in fresh shoot tissue was determined using the *in vivo* assay described for *Zostera marina*^58^. The *in vivo* technique was shown to yield consistently higher activity than the *in vitro* assay, which often gave negligible readings^15^. Extraction and assay for glutamine synthetase (GS) activity in new and fully extended leaf tissue was carried out following the method developed for *Z. marina*^25^, except that the incubation was carried out nearer to the aquaria temperature (30 °C).

To study carbon translocation, sucrose-phosphate synthase (SPS) from young but fully extended shoot tissue and sucrose synthase (SS) from the root–rhizome complex were extracted using a technique described in Brun *et al.*^27^ and assayed according to the protocol outlined in Zimmerman *et al.*^28^. The sucrose produced was quantified colorimetrically using anthrone assay^59^.

### Shoot and rhizome-root biochemistry

Shoot tissue nutrients (carbon and nitrogen) of ashed samples were analysed using an elemental analyser (Elementar Vario EL, Germany) interfaced to an isotope-ratio-mass-spectrometer (PDZ Europa 20–20, Sercon Ltd; Cheshire UK), as described in Takahashi *et al.*^45^. To study carbon storage, ground rhizome-roots samples were analysed for non-structural carbohydrates content according to procedure described in Collier *et al.*^35^. The summed amount of soluble carbohydrates and starch gave total non-structural carbohydrates (TNSC) content, expressed as milligrams dry weight^−1^ of tissue.

### Statistical analysis

Parameters were analysed using linear mixed effects models with *p*CO_2_ as a continuous predictor, and nitrate (ambient and enriched) as a categorical factor. Individual tanks were included as replicates, with sub-replicate pots nested within tanks. The nested factor was omitted for parameters without sub-replicate measurements (*T. hemprichii*: net primary production, respiration; both species: ^15^NO_3_^−^ uptake). For these parameters, measurements were terminated prematurely due to an unforeseen evacuation of the research station caused by a cyclone, and therefore the second sub-replicate could not be measured. Assumptions of normality and homogeneity of variances were tested with Shapiro-Wilks’ and Bartlett’s tests, respectively. Percentage data (%C and %N) were arcsine square-root transformed to meet the assumptions^60^. All statistical tests were assessed at α = 0.05 and analysed using R statistical software (R Development Core Team).

## Additional Information

**How to cite this article**: Ow, Y. X. *et al.* Nitrate fertilisation does not enhance CO_2_ responses in two tropical seagrass species. *Sci. Rep.* **6**, 23093; doi: 10.1038/srep23093 (2016).

## Supplementary Material

Supplementary Information

## Figures and Tables

**Table 1:** Experimental parameters.

*p*CO_2_ treatment	Nutrient	Measured parameters					Calculated parameters				Nutrient levels		
		DIC (μmol kg^−1^ SW)	A_T_ (μmol kg^−1^ SW)	pH(NIST)	Temperature(°C)	Salinity	*p*CO_2_ (μatm)	HCO_3_^−^ (μmol kg^−1^ SW)	CO_2_ (μmol kg^−1^ SW)	CO_3_^2−^(μmol kg^−1^ SW)	NH_4_^+^ (μΜ)	PO_4_^3−^ (μΜ)	NO_3_^−^(μΜ)
Control	−	1945.8 (9.59)	2234.8 (4.71)	8.01 (0.01)	28.47 (0.55)	34.74 (0.13)	435 (12.44)	1721.7 (13.66)	11.3 (0.42)	187.3 (2.45)	0.78 (0.37)	0.05 (0.02)	0.39 (0.25)
Control	+	1937.8 (9.49)	2233.8 (5.96)	8.02 (0.01)	28.53 (0.49)	34.74 (0.13)	422 (15.07)	1710.4 (14.13)	10.9 (0.43)	192.3 (2.02)	0.67 (0.32)	0.04 (0.02)	1.98 (0.34)
Intermediate	−	2045.8 (12.22)	2238.8 (5.25)	7.83 (0.04)	28.63 (0.76)	34.74 (0.13)	731 (78.43)	1875.5(21.95)	19.0 (2.09)	129.4 (13.06)	0.55 (0.18)	0.04 (0.02)	0.24 (0.13)
Intermediate	+	2047.4 (12.88)	2238.3 (4.76)	7.82 (0.05)	28.80 (0.72)	34.74 (0.13)	738 (88.59)	1877.4 (24.40)	19.1 (2.33)	131.0 (12.94)	0.60 (0.29)	0.04 (0.02)	1.80 (0.23)
High	−	2135.0 (18.95)	2240.4 (4.89)	7.63 (0.04)	28.70 (0.61)	34.74 (0.13)	1235 (129.49)	2001.1(22.17)	32.0 (3.15)	92.9 (5.91)	0.76 (0.55)	0.05 (0.02)	0.29 (0.16)
High	+	2130.6 (14.89)	2239.9 (5.20)	7.64 (0.04)	28.73 (0.58)	34.74 (0.13)	1190 (110.60)	1994.7 (18.18)	30.8 (2.67)	87.8 (4.85)	0.54 (0.18)	0.04 (0.02)	1.71 (0.68)

Values are given as mean ± S.D. Carbonate system parameters were calculated using measured values of total alkalinity (A_T_), total dissolved inorganic carbon (DIC), temperature and salinity on CO_2_calc software^52^.

**Table 2:** Linear mixed effects models for measured productivity response variables.

Parameter	Source	*Halodule uninervis*			*Thalassia hemprichii*		
		df	F	*p*	df	F	*p*
Net primary production	*p*CO_2_	1	4.669	0.049	1	0.184	0.675
	Nitrate	1	0.091	0.767	1	2.745	0.120
	*p*CO_2_ × Nitrate	1	0.721	0.410	1	3.648	0.077
Shoot respiration	*p*CO_2_	1	3.785	0.072	1	0.849	0.373
	Nitrate	1	5.199	0.039	1	0.226	0.642
	*p*CO_2_ × Nitrate	1	1.861	0.194	1	1.756	0.206
Rhizome-root respiration	*p*CO_2_	1	1.818	0.199	1	3.082	0.101
	Nitrate	1	8.593	0.011	1	0.584	0.458
	*p*CO_2_ × Nitrate	1	9.037	0.009	1	1.607	0.226
Growth rate (10 days)	*p*CO_2_	1	10.430	0.006	1	6.376	0.024
	Nitrate	1	3.418	0.086	1	1.427	0.252
	*p*CO_2_ × Nitrate	1	0.003	0.961	1	1.575	0.230
Growth rate (24 days)	*p*CO_2_	1	19.218	0.001	1	0.068	0.799
	Nitrate	1	1.014	0.331	1	0.870	0.367
	*p*CO_2_ × Nitrate	1	1.544	0.234	1	0.077	0.786
Sucrose phosphate synthase	*p*CO_2_	1	1.556	0.233	1	0.534	0.477
	Nitrate	1	5.109	0.040	1	0.436	0.520
	*p*CO_2_ × Nitrate	1	0.275	0.608	1	0.062	0.806
Sucrose synthase	*p*CO_2_	1	0.002	0.967	1	3.619	0.078
	Nitrate	1	1.677	0.216	1	0.389	0.543
	*p*CO_2_ × Nitrate	1	3.291	0.091	1	0.251	0.624
Total non-structural carbohydrates	*p*CO_2_	1	0.003	0.959	1	0.548	0.471
	Nitrate	1	0.053	0.821	1	0.994	0.336
	*p*CO_2_ × Nitrate	1	0.152	0.702	1	4.66 × 10^−4^	1.000

Variables were analysed with *p*CO_2_ as a continuous predictor and nitrate as a categorical factor. Individual aquarium tanks were included as replicates (N = 3), with two sub-replicate pots nested within aquaria. For net primary production, shoot and rhizome-root respiration, linear models were used for analysis, with aquaria as replicates (N = 3) and without nested sub-replicate pots. *P*-values < 0.05 are in bold.

**Table 3:** Linear mixed effect models for all nitrogen uptake and metabolism variables.

Parameter	Source	*Halodule uninervis*			*Thalassia hemprichii*		
		df	F	*p*	df	F	*p*
Nitrate uptake	*p*CO_2_	1	0.157	0.698	1	1.014	0.331
	Nitrate	1	0.000	0.984	1	0.156	0.698
	*p*CO_2_ ×Nitrate	1	0.380	0.548	1	7.392	0.017
Nitrate reductase	*p*CO_2_	1	3.076	0.101	1	1.144	0.303
	Nitrate	1	2.523	0.135	1	8.092	0.013
	*p*CO_2_ ×Nitrate	1	0.526	0.480	1	4.061	0.064
Glutamine synthetase	*p*CO_2_	1	0.142	0.712	1	0.089	0.769
	Nitrate	1	0.376	0.550	1	0.289	0.600
	*p*CO_2_ ×Nitrate	1	3.443	0.085	1	0.466	0.506
Carbon content	*p*CO_2_	1	1.420	0.253	1	0.928	0.352
	Nitrate	1	1.390	0.259	1	0.094	0.764
	*p*CO_2_ ×Nitrate	1	0.330	0.576	1	0.094	0.764
Nitrogen content	*p*CO_2_	1	0.310	0.584	1	2.180	0.162
	Nitrate	1	1.350	0.266	1	4.363	0.056
	*p*CO_2_ ×Nitrate	1	0.510	0.487	1	3.113	0.099
C:N ratio	*p*CO_2_	1	1.571	0.231	1	1.436	0.251
	Nitrate	1	3.227	0.094	1	4.846	0.045
	*p*CO_2_ ×Nitrate	1	0.282	0.604	1	3.258	0.093

Variables were analysed with *p*CO_2_ as a continuous predictor and nitrate as a categorical factor. Individual aquaria were included as replicates (N = 3), with two sub-replicate pots nested within aquaria. *P*-values < 0.05 are in bold.
