# Supplementary material for: Nitrate fertilisation does not enhance CO2 responses in two tropical seagrass species
Source: Sci Rep. 2016 Mar 15;6:23093. doi: 10.1038/srep23093 (PMC4792133; doi:10.1038/srep23093)
Supplement: Supplementary Information [file srep23093-s1.pdf]

Nitrate fertilisation does not enhance CO_2 responses in two tropical seagrass species

Ow Y.X.[1,2,4*], Vogel N.[2], Collier C.J.[1,3], Holtum J.A.M.[1], Flores F.[2] and Uthicke S.[2]

[1] College of Marine and Environmental Science, James Cook University, Townsville Queensland 4811, Australia

[2] Australian Institute of Marine Science, Townville MC Queensland 4810, Australia.

[3] Centre for Tropical Water & Aquatic Ecosystem Research (TropWATER), James Cook University, Cairns, Queensland 4870, Australia

[4] Experimental Marine Ecology Laboratory, Department of Biological Sciences, National University of Singapore, 14 Science Drive 4, Blk S3, #02-05, Singapore 117543

[*]Corresponding author (yan.ow@my.jcu.edu.au)

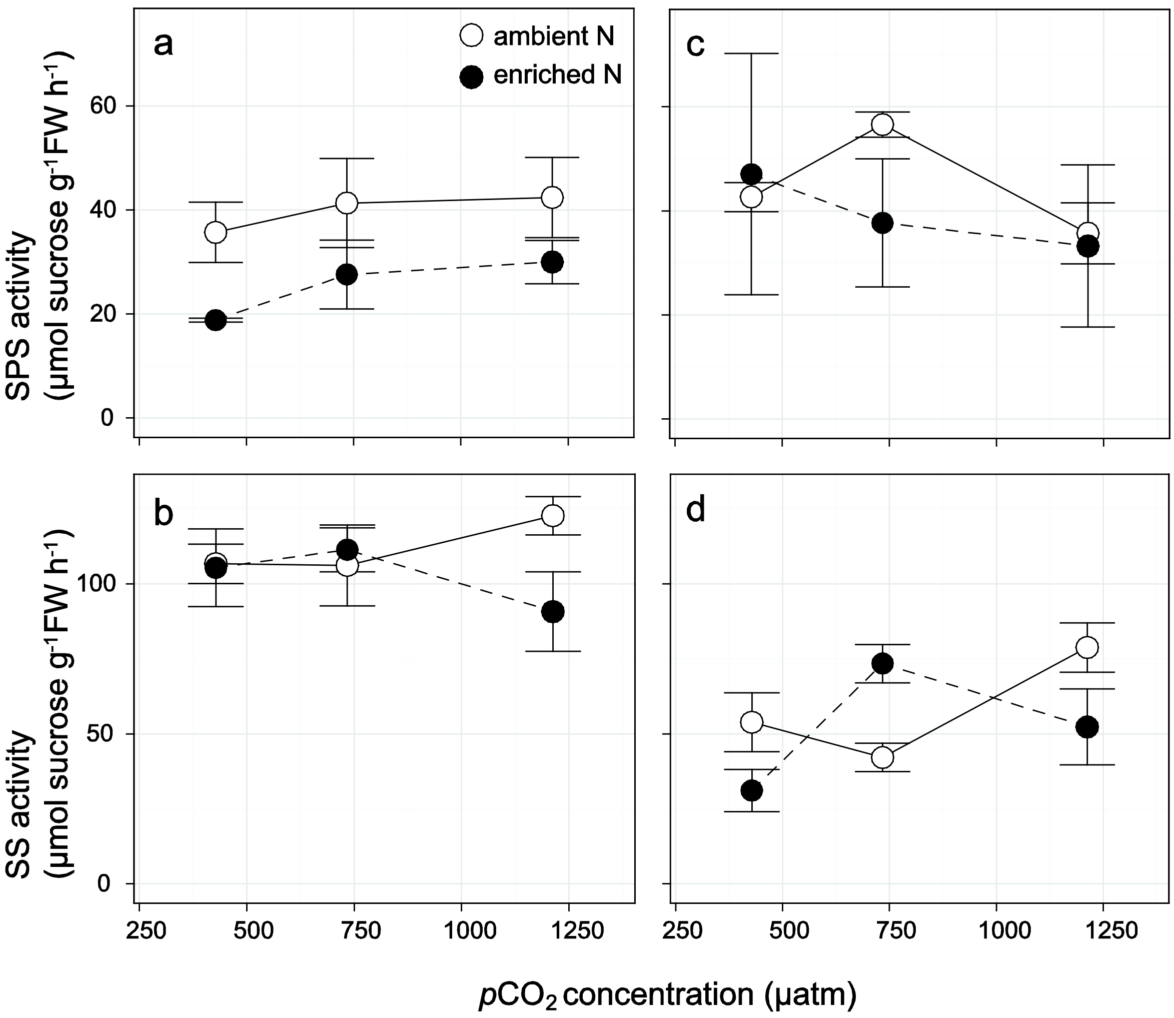

Supplementary Figure 1. Leaf sucrose-phosphate synthase (SPS) and rhizome sucrose synthase (SS) assayed from (a – b) *Halodule uninervis* and (c – d) *Thalassia hemprichii* across a range of pCO_2 concentrations. Values are average ± S.E. N = 3
